# Economic Performance of Oblique Lateral Lumbar Interbody Fusion (OLLIF) with a Focus on Hospital Throughput Efficiency

**DOI:** 10.7759/cureus.292

**Published:** 2015-07-30

**Authors:** Hamid Abbasi, Christopher M Murphy

**Affiliations:** 1 Tristate Brain and Spine Institute; 2 AMW Spine

**Keywords:** ollif, clinical economics, spinal fusion, reduced surgical time, reduced length of stay (los), reduced or time, minimally invasive spine surgery, healthcare cost reduction

## Abstract

Oblique lateral lumbar interbody fusion (OLLIF) is a minimally invasive lumbar surgery. Differences in resource consumption between open spinal surgeries, transformational lumbar interbody fusions (TLIF) and OLLIF, are not documented. We monetize quantifiable differences in resource utilization between the two procedures. A retrospective review of 124 surgeries was performed (OLLIF=69, TLIF=55). Standard conversion factors were used and values reported based on the levels (1-4) addressed at surgery. One level surgery time (OLLIF 62.9 vs. TLIF 134.9 minutes) and surgical expense (OLLIF $5,253 vs. TLIF $11,264) were reduced in the OLLIF population. Inpatient costs (OLLIF $5,712 vs. TLIF $9,271) and length of stay (LOS) were also reduced (OLLIF 2.6 vs. TLIF 4.2 days). Per case, reduced resource consumption suggests lower total hospital costs. Reduced surgical time and LOS can result in greater patient throughput per operating room and patient bed for OLLIF patients in hospitals that have resourced constrained environments.

## Introduction

Low back pain affects up to 80% of all people at some point during their lifetime and is one of the most expensive and prevalent health conditions in the Western world [1-2]. This condition is one of the most common causes of health care utilization, the leading cause of activity limitation for people under 45, and the third most common cause of surgical procedures in the United States [1].

The standard treatment for lower back pain, interbody fusion, in an invasive procedure that requires stripping the muscles and soft tissue. This leads to increased blood loss and a long recovery time. Harms and Rolinger developed the transformational lumbar interbody fusion (TLIF) as a treatment for disc disorders in 1982 [3]. However, during the TLIF procedure, muscles are detached and denervated which may cause significant morbidity [4]. To address these issues, minimally invasive (MI) TLIF was developed [5]. While MI TLIF has been shown to decrease blood loss and complication rates relative to open TLIF, surgery times and long-term outcomes have been reported to be similar [6-8].

Oblique posterior lateral lumbar fusion (OLLIF) is a surgical procedure designed for a minimally invasive spinal fusion [9-11]. The OLLIF procedure allows for fusion of the lumbar spine through a single 10-15 mm incision, with faster surgery times and easier approach than any previous technique. This procedure is normally performed for patients that require a spinal fusion but do not want the recovery time required in a traditional spinal fusion surgery.

In recent years, the rate of disability due to low back pain has increased dramatically, and consequently, costs have skyrocketed [1, 12]. As such, advancements in the surgical treatment of lower back pain could benefit numerous patients annually and contribute to lower health care costs. In this article, we present perioperative outcome data from 69 OLLIF procedures, compare them to 55 open TLIFs on 125 levels done by the same surgeon, and monetize quantifiable differences in the resource utilization between the two procedures.

## Materials and methods

### Study design

This was a retrospective case series including 69 OLLIF patients and 55 open TLIF controls. The exempt status of this study, in accordance with FDA 21 CFR 56.104 and DHHS 45 CFR 46.101 regulations, was approved by the Pearl Institutional Review Board (15-TRIS-101; Indianapolis, IN 46225) in February 2015.

### Surgical procedures

Surgical procedures were performed as previously described [9-11]. All procedures were completed by the same surgeon as single surgeon procedures. Patient surgical indications are listed shown below (Table 1). To eliminate selection bias, the TLIF control group was selected from patients who underwent surgery before the surgeon started performing OLLIF. All 124 procedures were performed in two Minnesota hospitals: Douglas County Hospital, 111 17th Ave E, Alexandria, MN and Riverview Health, 323 Minnesota St, Crookston, MN. Surgeries were performed between March 2012 and December 2013. The study size derives from the number of surgeries accomplished in this time frame.

**Table 1:** Patient surgical indications

Indication	n
Degenerative disk disease	61
Disk herniation	14
Listhesis	22
Stenosis	15
Scoliosis	2

### Outcome measures

Anesthesia/surgery times and blood loss were recorded for all patients by clinic staff and entered into the EMR database immediately after surgery. Because no suction is used in OLLIF procedures, blood loss for the OLLIF group was measured by weighing sponges and subtracting dry weight. To monetize the cost per minute for an operating room (OR) per case and for an average hospital day, a published reference for this amount was identified in the medical literature and adjusted by using consumer price index for medical costs [13]. These values were reported both in aggregate and stratified based on the number of levels they had addressed at the time of their surgery (one, two, three or four).

Data were collected from the clinic electronic medical record (EMR) and summarized in Microsoft Excel.

Mann-Whitney U-tests were utilized to test the null hypothesis that the OLLIF and TLIF groups have the same or identical mean distributions for age, BMI, blood loss, and the uncensored time duration variables.  All data analyses were performed using SPSS (IBM SPSS Statistics for Windows, Version 22.0. Armonk, NY: IBM Corp.).

## Results

Summary statistics for the two study groups are shown below (Table 2). There were no significant differences between the groups in either BMI or age. The only exception was that OLLIF three level patients were significantly older than their counterparts in the TLIF comparison group.

**Table 2:** Mean summary statistics of the study groups

1 Level	OLLIF	TLIF	p value
Number	28	9	-
Age	56.1±15.2	64.1±20.9	0.226
BMI	29.5±5.8	31.0±4.8	0.541
2 Level	OLLIF	TLIF	p value
Number	24	19	-
Age	59.0±18.5	57.3±15.4	0.797
BMI	30.5±4.8	30.8±6.5	0.973
3 Level	OLLIF	TLIF	p value
Number	12	14	-
Age	68.7±11.7	58.3±8.7	0.023 **
BMI	30.9±8.4	35.4±6.3	0.160
4 Level	OLLIF	TLIF	p value
Number	4	9	-
Age	63.0±15.5	68.8±17.6	0.604
BMI	30.0±8.3	30.6±8.2	0.625

Perioperative outcomes are shown below (Table 3). In all groups, OLLIF significantly reduced surgery times, blood loss, and hospital stay compared to TLIF. There was one exception in that there was no significant difference between the two groups in the length of hospital stay in the three level patient groups. In the one level group, mean blood loss was reduced almost 11-fold (p<0.001). In general, blood loss per patient was less in OLLIF when compared with TLIF (Table 3). Blood loss volumes were 33.9 mL and 355 mL for one level surgery (p<0.001), 55 mL and 452 mL for two level surgery (p<0.001), 94 mL and 618 mL for three level surgery (p<0.001), and 102 mL and 589 mL for four level surgery (p=0.009), for OLLIF and TLIF, respectively. The differences in per procedure blood loss were 321 mL for one level surgery, 397 mL for two level surgery, 524 mL for three level surgery, 487 mL for four level surgery and 448 mL overall.

**Table 3:** Mean perioperative outcomes of the study groups

1 Level	OLLIF	TLIF	p value
Blood loss (ml)	33.9±17.2	355.0±131.5	<0.001
Surgery time (min)	62.9±29.4	134.9±21.7	<0.001
Days to discharge	2.6±1.7	4.2±1.2	0.001
2 Level	OLLIF	TLIF	p value
Blood loss (ml)	55.4±78.4	452.6±327.6	<0.001
Surgery time (min)	70.5±42.0	175.3±39.3	<0.001
Days to discharge	3.3±1.1	5.8±6.5	0.010
3 Level	OLLIF	TLIF	p value
Blood loss (ml)	94.2±57.6	618.6±353.9	<0.001
Surgery time (min)	114.2±42.9	213.7±40.5	<0.001
Days to discharge	3.2±2.2	4.3±1.3	0.940
4 Level	OLLIF	TLIF	p value
Blood loss (ml)	102.3±66.1	589.4±255.3	0.009
Surgery time (min)	161.8±25.8	250.2±73.6	0.020
Days to discharge	4.6±0.6	6.7±1.6	0.011

Resource utilization observed in the OLLIF and TLIF surgeries are presented below (Table 4), first matched for the number of levels and then overall. The average value for OLLIF is presented followed by the average value for TLIF; the percentage is the percent of the TLIF value represented by the OLLIF value. Across all surgeries studied, surgical time for the OLLIF surgeries was 41.6% of that seen with TLIF surgeries (Table 3-4; 79.9 vs. 191.9 minutes). The shorter surgical time for OLLIF surgeries remained consistent when surgeries were stratified and matched for the same number of levels involved (62.9 vs. 134.9 for one level, 70.5 vs. 175.3 for two levels, 114.2 vs. 213.7 for three levels, and 161.8 vs. 250.2 for four levels). Overall, when surgical time was converted to an operating cost of the hospital, using a standard conversion factor updated for inflation, the difference in cost of surgery attributable to surgical time was $6,671 for OLLIF vs. $16,029 for TLIF.

**Table 4:** Resource utilization observed in the OLLIF and TLIF surgeries

Levels	Surgical Time (min) OLLIF/TLIF	Surgical Time Costs ($) OLLIF/TLIF	LOS (Days) OLLIF/TLIF	Inpatient Costs ($) OLLIF/TLIF
1	62.9/134.9 (46.6%)	$5,253/$11,264 (46.6%)	2.6/4.2 (61.9%)	$5,712/$9,271 (61.6%)
2	70.5/175.3 (40.2%)	$5,884/$14,640 (40.2%)	3.2/5.8 (56.9%)	$7,030/$12,830 (54.8%)
3	114.2/213.7 (53.4%)	$9,538/$17,847 (53.4%)	3.2/4.3 (74.4%)	$6,986/$9,403 (74.3%)
4	161.8/250.2 (64.7%)	$13,512/$20,896 (64.7%)	4.6/6.7 (68.6%)	$10,106/$14,654 (68.9%)
Overall	79.9/191.9 (41.6%)	$6,671/$16,029 (41.6%)	3.1/5.3 (58.5%)	$6,701/$11,583 (57.8%)

Overall, across all surgeries studied, LOS for OLLIF surgeries was 58.5% of that seen with TLIF surgeries (3.1 vs. 5.3 days). The trend of shorter LOS for OLLIF surgeries remained consistent when surgeries were stratified and matched for the same number of levels involved (2.6 vs. 4.2 for one level, 3.2 vs. 5.8 for two levels, 3.2 vs. 4.3 for three levels, and 4.6 vs. 6.7 for four levels). Overall, when LOS was converted to inpatient operating costs of the hospital, the difference in cost of surgical admission was $6,701 for OLLIF vs. $11,583 for TLIF.

## Discussion

MI surgical techniques are available for treating a wide range of clinical indications in the lumbar spine. In general, clinical outcomes following MIS procedures compare favorably to traditional open surgery [14-16]. OLLIF has been described as the first MI fusion that is faster than open surgery and has been found by the authors to overcome difficulties characteristic of traditional open fusions, thereby, making it a safe and reliable alternative to open or MI TLIF [9-11] (unpublished data).

While these and other data suggest that minimally invasive spine surgery reduces morbidity, hospital stay, and accelerates a patient’s rehabilitation time; data regarding cost effectiveness of MI techniques are limited. Less than 1% of the evaluations on lumbar spine arthrodesis procedures published from 2004 to 2009 contain a cost-effectiveness analysis [17]. There are even fewer data on the benefits of MI surgeries. This study monetized quantifiable differences in resource utilization between two MI procedures, OLLIF and TLIF.

In this single-surgeon case series of 69 consecutive OLLIF surgeries and 55 consecutive open TLIF controls, the use of two key hospital resources were decreased in the OLLIF population by approximately one-half over the TLIF population (42% for surgical/OR time and 58% for LOS). Mean blood loss was also reduced in the OLLIF population. In general, one unit of red blood cells is approximately 500 mL; in this study, the differential blood loss between the OLLIF and TLIF procedures was approximately 0.9 units of blood per patient. However, while the use of the OLLIF procedure could easily have triggered the differential utilization of blood products since actual transfusion data were not captured, this amount could not be monetized.

Using a retrospective patient cohort for comparison might bias the study data as clinical practices may change over time. However, data accumulated on perioperative measures, such as blood loss and OR time, were collected almost completely and clearly shows that OLLIF improves on TLIF. Due to the magnitude of this change, it is unlikely to be just a side-effect of the study design or this particular surgeon’s skill.

Therefore, OLLIF justifies further study as it has the potential to significantly improve the outcomes of patients with lumbar fusions. This study further identifies a need for high-quality cost-effectiveness studies comparing open lumbar spine surgeries using MI surgical techniques. Ongoing prospective, multi-centered studies are warranted to describe the resource consumption further.

## Conclusions

The cost reductions and faster recovery times associated with the OLLIF procedure make it an appealing alternative to the traditional open fusions available for patient and insurance providers. The reduction in the use of these key hospital resources suggests that hospitals that are constrained by OR or hospital bed availability may be able to achieve greater throughput efficiency by increasing the overall percentage of patients receiving the OLLIF surgery.

## References

[1] Andersson GB (1999). Epidemiological features of chronic low-back pain. Lancet.

[2] Frymoyer JW, Cats-Baril WL (1991). An overview of the incidences and costs of low back pain. Clin North Am.

[3] Harms J, Rolinger H (1982). A one-stage procedure in operative treatment of spondylolisthesis: Dorsal traction-reposition and anterior fusion (author’s translation) (article in German). Z Orthop Ihre Grenzgeb.

[4] Sihvonen T, Herno A, Paljärvi L, Airaksinen O, Partanen J, Tapaninaho A (1993). Local denervation atrophy of paraspinal muscles in postoperative failed back syndrome. Spine.

[5] Ozgur BM, Yoo K, Rodriguez G, Taylor WR (2005). Minimally invasive technique for transforaminal lumbar interbody fusion (TLIF). Eur Spine J.

[6] Parker SL, Lerner J, McGirt MJ (2012). Effect of minimally invasive technique on return to work and narcotic use following transforaminal lumbar inter-body fusion: a review. Prof Case Manag.

[7] Shunwu F, Xing Z, Fengdong Z (2010). Minimally invasive transforaminal lumbar interbody fusion for the treatment of degenerative lumbar diseases. Spine.

[8] Goldstein CL, Macwan K, Sundararajan K, Rampersaud YR (2014). Comparative outcomes of minimally invasive surgery for posterior lumbar fusion: A systematic review. Clin Orthop Relat Res.

[9] Kambin P, Sampson S (1986). Posterolateral percutaneous suctionexcision of herniated lumbar intervertebral discs. Report of interim results. Clin Orthop Relat Res.

[10] Kambin P, Zhou L (1997). Arthroscopic discectomy of the lumbar spine. Clin Orthop Relat Res.

[11] Park JW, Nam HS, Cho SK, Jung HJ, Lee BJ, Park Y (2011). Kambin’s triangle approach of lumbar transforaminal epidural injection with spinal stenosis. Ann Rehabil Med.

[12] Luo X, Pietrobon R, Sun SX, Liu GG, Hey L (2004). Estimates and patterns of direct health care expenditures among individuals with back pain in the United States. Spine.

[13] Macario A (2010). What does one minute of operating room time cost?. J Clin Anesth.

[14] Cummock MD, Vanni S, Levi AD, Yu Y, Wang MY (2011). An analysis of postoperative thigh symptoms after minimally invasive transpsoas lumbar interbody fusion. J Neurosurg Spine.

[15] O’Toole JE, Eichholz KM, Fessler RG (2009). Surgical site infection rates after minimally invasive spinal surgery. J Neurosurg Spine.

[16] Villavicencio AT, Burneikiene S, Roeca CM, Nelson EL, Mason A (2010). Minimally invasive versus open transforaminal lumbar interbody fusion. Surg Neurol Int.

[17] Rihn JA, Berven S, Allen T, Phillips FM, Currier BL, Glassman SD, Nash DB, Mick C, Crockard A, Albert TJ (2009). Defining value in spine care. Am J Med Qual.

